# “Long Overdue”: Nurse and Resident Physician Perspectives on Implementation of Dual-Handset Interpreter Phones in the Inpatient Setting

**DOI:** 10.1089/heq.2022.0023

**Published:** 2023-02-16

**Authors:** Maria E. Garcia, Sunita Mutha, Anna M. Napoles, Lev Malevanchik, Mia Williams, Leah S. Karliner

**Affiliations:** ^1^ Department of Medicine, Center for Aging in Diverse Communities, University of California, San Francisco, San Francisco, California, USA.; ^2^ Multiethnic Health Equity Research Center, Division of General Internal Medicine, Department of Medicine, University of California, San Francisco, San Francisco, California, USA.; ^3^ Department of Epidemiology and Biostatistics, Partnerships in Research in Implementation Science for Equity (PRISE) Center, University of California, San Francisco, San Francisco, California, USA.; ^4^ Division of General Internal Medicine, Department of Medicine, University of California, San Francisco, San Francisco, California, USA.; ^5^ Division of Intramural Research, National Institute on Minority Health and Health Disparities, National Institutes of Health, Bethesda, Maryland, USA.; ^6^ Division of Hospital Medicine, Department of Medicine, University of California, San Francisco, San Francisco, California, USA.

**Keywords:** hospital settings, interpreters, language barriers, limited English proficiency, remote interpretation

## Abstract

**Background:**

Patients with language barriers suffer significant health disparities, including adverse events and poor health outcomes. While remote language services can help improve language access, these modalities remain persistently underused. The objective of this study was to understand clinician experiences and challenges using dual-handset interpreter telephones and to inform recommendations for future language access interventions.

**Methods:**

We conducted four focus groups with nurses (*N*=14) and resident physicians (*N*=20) to understand attitudes toward dual-handset interpreter telephones in the hospital, including general impressions, effects on communication, situations in which they did and did not use them, and impact on clinical care. Three researchers independently coded all transcripts using a constant comparative approach, meeting repeatedly to discuss coding and to reconcile differences to reach consensus.

**Results:**

We identified five salient themes, including increased language access (improved convenience, flexibility, and versatility of phones over in-person or *ad hoc* interpreters); effects on interpersonal processes of care (improved ability to communicate directly with patients); effects on clinical processes of care (improvements in critical patient care functions, including pain and medication management); impact on time (needing extra time for interpreted encounters and perceived delays impacting future use); and patients for whom, and circumstances in which, the dual-handset interpreter telephone is inadequate (e.g., complex discussions, hands-on instruction, or multiple speakers are present).

**Conclusions:**

Our findings indicate that clinicians value dual-handset interpretation in bridging communication barriers and highlight recommendations to guide future implementation interventions to increase the uptake of remote language services in hospital settings.

## Introduction

Almost 68 million individuals in the United States speak a language other than English at home and nearly 38% of these individuals speak English less than very well or have limited English proficiency (LEP).^1^ The United States has seen a rise in the number of individuals with LEP in recent decades, presenting a challenge for health care systems that must overcome language barriers to provide high-quality patient-centered care.^2,3^

Patients with LEP experience substantial health disparities in processes and outcomes of care, including decreased comprehension of their diagnoses, decreased care satisfaction, poorer adherence, and increased medication complications compared with English speakers.^4–9^ When admitted in the hospital and professional interpreters are not used, individuals with LEP experience more adverse events, potentially longer stays, higher 30-day readmissions, and higher sepsis mortality compared with English speakers.^3,6,10–14^

The Agency for Healthcare Research and Quality (AHRQ) identified three common causes of errors leading to adverse events for patients with LEP: (1) use of *ad hoc* (untrained family or staff) interpreters, (2) clinician use of basic language skills to “get by,” and (3) provider unawareness of patient cultural beliefs and traditions that affect care delivery.^3^ Use of professional interpreters, whose services improve patient–physician communication, can address these root causes of adverse events for patients with LEP. Furthermore, professional interpreters enhance communication, appropriate resource use in clinical care, and patient and clinician satisfaction.^7^

Despite federal regulations and Joint Commission Standards requiring professional interpreters or language-concordant clinical care of patients with LEP, access to and use of professional interpreters are often limited.^3,6,12,15–19^ Multifaceted interventions to promote remote language services improve use of professional interpreters, but significant gaps persist; documented use of interpreters often remains below 40% of hospital and emergency room encounters for patients with LEP.^16,17,20^

For hospitalized patients, the lack of access to language services is compounded by the frequent, unpredictable, and brief nature of interactions; time pressures; limited availability and complex scheduling for in-person interpreters; and the 24-h nature of hospital care.^21^ These factors may lead to delays in clinical evaluation of patients with LEP while awaiting an interpreter.^6,21^ Access to remote interpretation through telephone or video may ameliorate these delays.^16^

The COVID-19 pandemic, with restrictions on the number of individuals entering patient rooms, created new barriers to in-person interpreter access and increased the need for uptake of remote language services.^22^ Clinicians—nurses and physicians—often drive access to interpreters for patients who prefer a non-English language; therefore, a better understanding of barriers and facilitators to clinician use of dual-handset interpreter telephones, a widely available and enduring remote language service modality in hospitals, may enable targeted interventions of this and other modalities to improve language access.

We conducted focus groups with nurses and resident physicians in a tertiary care hospital, which had implemented on-demand access to professional interpreters through dual-handset telephones in patient rooms. This intervention did increase utilization of professional interpreters, but had mixed results on clinical outcomes, demonstrating some persistent gaps in adequate use of professional interpreters.^8,12,20^

To better understand dual-handset interpreter telephone use and remaining implementation challenges, we elicited focus group participants' experiences with and perceptions of dual-handset interpreter telephones, factors influencing use (including types of clinical interactions most and least suited to use of dual-handset telephones), and their effect on communication and clinical care.

We report the findings from those focus groups and implications for future interventions intended to increase use of professional interpretation in hospital settings.

## Methods

### Description of the dual-handset interpreter telephone intervention

The bedside interpreter intervention has been previously described.^8,12,20^ It consisted of placing a dual-handset interpreter telephone at the bedside of every patient who preferred a non-English language for their health care starting in late December 2012 in one academic health center. A programmed button provides rapid 24-h access to professional medical interpreters for more than 100 languages.

The dual-handset allows the patient to speak into one handset, the clinician to speak into the second handset, and the professional interpreter (vendor-based or in-house staff) to facilitate the conversation from a remote location. Before the intervention, up to three dual-handset interpreter telephones were available on most units, located on mobile carts, and kept at the nursing station or in locked cabinets until needed.

In-person professional interpreters could be scheduled during weekdays (8 am to 5 pm) before and throughout the intervention period.

### Data collection

We conducted four focus groups, two with nurses and two with resident physicians, in January 2015 to understand their experiences with implementation of the dual-handset interpreter telephones in the hospital. Using an open-ended guide, participants were asked about their general attitudes toward the dual-handsets, what they liked and disliked about them, the effects on communication with patients, situations in which they did and did not use them, and their impact on clinical aspects of providing care.

We recruited a purposive sample of nurses and resident physicians from inpatient settings. All nurses and resident physicians who had worked in the hospital after implementation of the dual-handset interpreter telephones were eligible to participate in the study. We also targeted attending physicians working in the hospital, but none chose to participate due to time constraints. Nurses were recruited through fliers posted in the nurses' lounges and work mailboxes and sent out in floor-based newsletters.

Similarly, resident physicians were recruited through e-mail listservs and fliers posted in the residents' lounges and charting areas and placed in work mailboxes. An experienced facilitator led each focus group (A.M.N. or S.M.), with assistance from a second investigator on the team (L.S.K., A.M.N., or S.M.) who took detailed notes. Focus groups lasted up to 90 min and were audio-recorded and transcribed verbatim.

Due to a recorder malfunction, there was no transcript for one focus group; however, the investigator in attendance took detailed notes, including verbatim quotes that were included in the analyses. Nurses and resident physicians (hereafter “physicians”) completed a brief demographic questionnaire after the focus group and received $100 for participating.

Consent forms and the study protocol were approved by the UCSF Institutional Review Board.

### Data analysis

Three experienced qualitative researchers (S.M., A.M.N., and L.S.K.) independently coded transcripts and the extensive notes from the fourth focus group, using a constant comparative approach (comparing as many similarities and differences as possible in the data to generate coding categories and their definitions).^23^ The qualitative data analysis was conducted using QSR NUD*IST software, which facilitated analysis on multiple levels of coding, overlapping codes, and nested responses.

Coders met repeatedly to discuss their coding and reconcile differences until consensus was reached on the coding categories, definitions, and results. Demographic questionnaire data were analyzed using descriptive statistics.

## Results

### Participant characteristics

There were 34 focus group participants. The majority of nurses were women; physicians included about equal numbers of men and women (Table 1). Most of the participants were Asian or white. Nurses were less likely than physicians to report speaking a language other than English. Similar proportions of nurses and physicians reported having family members with LEP.

**Table 1.** Demographics of Focus Group Participants (Nurses and Resident Physicians; *N*=34)

	Nurses, *N*=14	Physicians, *N*=20
	*N* (%)	*N* (%)
Women	13 (93)	9 (45)
Race/ethnicity		
Latino/a/x	1 (7)	0
Asian	6 (43)	10 (50)
White	7 (50)	8 (40)
Mixed	0	2 (10)
Proficiency in non-English language		
Not at all	3 (21)	2 (10)
Poor	5 (37)	6 (30)
Fairly well	1 (7)	5 (25)
Well	3 (21)	5 (25)
Very well	0	2 (10)
Missing	2 (14)	0
Family members with limited English proficiency	5 (36)	8 (40)

### Perspectives of the dual-handset interpreter telephones

We identified five themes related to the implementation of dual-handset language services (see Table 2 for representative quotes), including increased language access, effects on interpersonal processes of care, effects on clinical processes of care, impact on time, and circumstances where dual-handset interpreter telephone use is inadequate. Each theme is discussed below.

**Table 2.** Themes and Illustrative Quotes from Focus Groups with Nurses (*N*=14) and Resident Physicians (*N*=20)

Theme	Description	Illustrative quotes
Increased language access	Increases language access due to: placement of dual-handsets in every room, environmental cue to physician or nurse to use language services, on-demand availability, flexibility to use for interactions of varying duration, and accommodating many languages.	“Long overdue. Now they're in every room, whereas there used to only be one or two on a unit and always hard to find.” (Nurse) “Having the phone in the room—it's just another cue, “Oh, you know what? I really need to get on with interpreter services.” So, either I'm gonna use that phone, or I'm gonna schedule somebody, ‘cause we really have a major teaching thing, more than I can do on the phone.” (Nurse) “… with an [in-person] interpreter you have to call them. You have to wait, and then so you have to schedule your whole day around it, whereas the interpreter phone you can just use right away.” (Nurse) “Now I use it for even one minute, two minutes, however long it may take.” (Nurse) “Definitely workflow issues, but it's kind of amazing that I can call up a Toishanese interpreter at 3 in the morning and have a conversation [with a patient].” (Physician)
Subtheme: Provider-centered tool	Perceived as a provider-initiated tool rather than a tool that is equally available to patients.	“It's a very one-way utilization pattern. Like we [providers] get the phone when we need it … It's our tool. It doesn't help [patients] necessarily communicate with us on their time or initiatives.” (Physician) “I came in on day shift, and they told me that the patient was fine all night. And then when I used the phone, they [the patient] were like, ‘Oh, I've been nauseous, and I didn't sleep all night, and I was in pain all night.’ So, they were quiet all night. Just quiet and miserable. And I asked them, ‘Why didn't you ask for an interpreter?’ And they're like, ‘I didn't know you had it.’” (Nurse)
Effects on interpersonal processes of care	Use of telephones: enhances direct verbal communication, improves the ability to transcend cultural stereotypes, results in fewer assumptions, improves the ability to establish rapport, and elevates the importance of clinician–patient interaction.	“But when you have the handheld set, you can actually look at the patient and you each are speaking to each other. And so, that is—I mean it's a subtlety, but—an important feature that these phones—this manner of interpreter phone has. That it prioritizes doctor-patient interaction a bit more.” (Physician) “Yeah, sometimes I feel like we might stereotype certain cultures, too. So, you'll be thinking, ‘This little Chinese lady probably won't want to take morphine and whatnot,’ but in fact, she may have been taking morphine at home all along. You know? So, you would just be like, ‘Oh, she probably doesn't want this. She just wants hot water.’ Things like that.” (Nurse)
Effects on clinical processes of care	Telephone use improves: pain assessment and management, assessment of symptoms, evaluation of confusion/delirium, medication management, and discharge process.	“I think they're really helpful for pain management … and that really is a conversation … It's really important to explain the whole nuance of pain medication [to say], ‘Hey, if this isn't working, we can change it …’ I do need that phone to [say], ‘Really, what's going on with you for your pain?’ And, ‘Really, this is what we can offer you …’ So, I think for the pain piece, it's really invaluable.” (Nurse) “It gives you an opportunity to explain what medication you're giving them, and what it's for. And, it allows them to tell you, ‘Oh, yeah, I stopped taking that medication a few weeks ago. I don't know why they're giving it to me here.’ Without it, otherwise, there would be none of that.” (Nurse) “So, when that person looks like they are having that chest pain, I'm not waiting for their mom to show up. I'm not waiting for a Spanish-speaking PCA [patient care assistant] who's not on her lunch break. That phone's coming out.” (Nurse)
Impact on time	Use imposes a burden under time pressure (i.e., pre-rounds), or due to wait time, or pace of care.	“I feel like it's a lottery—how long will it take until someone answers the phone?” (Physician) “I never use the interpreter phone on pre-rounds. Ever…because … there's just not time, and you can get a clinical—just all based on their values and their vitals—with the expectation that I'll go back and see them later, when I have more time.” (Physician) “The times [when I don't use the phones] are frustrating … when there are a lot of external pressures and then I don't feel like I'm being the doctor I want to be or communicate the way that I typically am able to.” (Physician) “I just only wanted to ask one question, but the patient was saying a lot of things, and I didn't know how to hang up at times, ‘cause they feel really happy that they're speaking to someone of their language, and then they start telling their whole life story.” (Nurse)
Circumstances where the dual-handset interpreter telephone is inadequate	Use is inappropriate in certain circumstances (e.g., multiple speakers, family meetings, procedures, complicated or complex teaching, or discharge instructions) or with patients with certain conditions (e.g., hearing loss, cognitive impairment, poor hand dexterity/functioning).	“Usually [use] the handset, but sometimes, especially if there's a lot of family members that have a lot of questions about how to take care of the family member. And then it's not just teaching one person; it's teaching everybody. Then it's so much easier to have someone in person, and the interpreter [via handset phone] doesn't even know who they're talking to, ‘cause there's three or four different voices. And it's—then people are like—all sort of talking, and they just interrupt, and nobody knows what's going on.” (Nurse) “I definitely felt like the intern and the patient were able to have a more face-to-face interaction. But then it sort of left the rest of the team out of the whole conversation. And then when we would put the speaker phone on, the patient got very confused and didn't know where to look, didn't know who he was talking to.” (Physician) “The main issue I've had with them is really with patients that have difficulty hearing. And that actually comes up relatively frequently, especially in elderly patients that we often see on the medicine service.” (Physician)

#### Increased language access

Participants viewed the convenience, flexibility, and versatility of the dual-handset interpreter telephones as an improvement over relying on in-person or *ad hoc* interpreters only. The availability of dual-handsets “on-demand” eliminated the need to schedule an interpreter in advance and both nurses and physicians could fit them into their workflow rather than having to accommodate an interpreter's availability.

Flexibility to use the handsets for different types of communication (e.g., brief check-ins to lengthy exchanges) was viewed positively, compared with situations where participants might feel pressured to end a conversation because the in-person interpreter was needed elsewhere. Participants emphasized the increased language access and convenience of having a dual-handset telephone in every room.

However, they also noted that dual-handsets were mostly a provider-centered tool, in that only the health care team initiated use, and patients were often unaware that they could request an interpreter.

#### Effects on interpersonal processes of care

Participants expressed an improvement in their ability to communicate directly with patients, rather than having to rely on vital signs, nonverbal cues, guesses, or assumptions. With increased access to interpreters, they felt more able to establish rapport with patients and to afford greater attention to the clinician–patient interaction.

They also perceived that the ability to ask patients questions about their health-related beliefs and attitudes, rather than relying on cultural stereotypes, helped bridge cultural differences.

#### Effects on clinical processes of care

Participants reported improvements in several critical patient care functions because of their ability to easily communicate directly with patients. These functions included the ability to more comprehensively assess and manage patients' pain, obtain an accurate picture of patients' symptoms and mental status, and better manage medications.

Participants commented that dual-handsets allowed patients to more fully engage in their care, for example, by facilitating a more robust conversation about patients' pain experiences and options for managing it. Similarly, participants could more fully clarify inconsistencies in medication use and explain their purpose.

#### Impact on time

Despite the increased ease of access, participants remarked on needing extra time for interpreted conversations and occasionally noted greater delays in obtaining a remote interpreter under specific circumstances (e.g., early mornings, weekends, less common languages). While perceived delays were infrequent, participants noted that delays negatively affected their subsequent decisions to use dual-handsets.

As a result, in circumstances where time pressures were perceived as especially great, handset use was viewed as a burden (e.g., during physician “pre-rounds,” the daily morning practice of seeing patients to discuss overnight events and gather information on current clinical status before rounds with the attending physician).

#### Circumstances where the dual-handset interpreter telephone is inadequate

Participants reported circumstances in which the dual-handset telephones were inappropriate and other modes of interpretation were necessary. These circumstances included complex discussions or hands-on instruction, such as when planning for hospital discharge. The presence of multiple speakers, such as in family meetings or on team rounds, also made it difficult to use the dual-handset telephones.

While the speaker function was an option, its use could lead both to patient and interpreter confusion about who was speaking and to frequent interruptions. Additionally, the dual-handset telephones were difficult to use with patients with limited use of their hands or who had hearing loss or cognitive impairment.

## Discussion

This qualitative study explored nurses' and physicians' experiences with and perceptions of using dual-handset interpreter telephones and the impact on clinical care. Participants noted increased convenience, flexibility, and versatility offered by implementation of this service in the hospital. Dual-handset interpreter telephones were felt to improve clinician ability to establish rapport with patients and enable direct communication—with less reliance on nonverbal cues or family members—for clinical assessments throughout the hospital stay.

The ubiquitous nature of the dual-handset telephones increased access to professional interpreters, particularly for those brief interactions in which participants may have previously tried to “get by” without an interpreter.

We identified several areas for improvement when designing future interventions to increase language access for hospitalized patients with non-English language preference. Participants viewed delays in connecting with an interpreter as particularly problematic, especially when time pressures were high; importantly, these perceived delays were felt to impact subsequent use of remote language services.

Certain patient factors, such as hearing loss or cognitive impairment, were identified as needing additional strategies to bridge the communication barrier. Participants noted that while dual-handset telephones provided direct communication, they were a less adequate replacement for in-person interpreters in certain circumstances. In particular, they identified intensive educational or high-stakes conversations such as discharge planning or goals of care discussions as better served by in-person interpretation.

Our study has some limitations. It was conducted in a single academic health system and focused on the use of one modality of remote language services (dual-handset interpreter telephones) by resident physicians and nurses only. It is unclear whether the findings presented here reflect the experiences of others such as attending physicians or other members of care teams who often do hands-on complex patient teaching (e.g., respiratory and physical therapists, dieticians).

Furthermore, rapid development and adoption of communication technologies (e.g., video-mediated interpretation) since the time of our data collection, may limit the relevance of dual-handset telephone interpretation. However, during 2020, 52% of interpreted encounters used remote telephone interpretation in this study hospital.

One of the few studies evaluating interpreter use during the COVID-19 pandemic similarly found that video-only interpretation decreased from 56% to 17% and telephone interpretation increased from 18% to 81% in the emergency department,^24^ demonstrating the persistence of this technology.

While video-mediated interpretations are now more widely available, for rare languages or during times of high demand, telephone interpretation remains the backup modality. Furthermore, the lessons learned in our study are likely quite relevant for delivery of remote language services in general and may apply to health care settings where limited resources preclude implementation of the latest technologies.

Access to professional interpreters is essential for effective communication and high-quality care for patients who prefer a non-English language.^3,6,7^ Many hospitals throughout the country continue to have difficulties bridging language barriers and many hospitalized patients who prefer a non-English language continue to have limited access to professional interpreters and must rely on *ad hoc* or family members for communication; this is an obstacle to equitable care that has been further compounded by the COVID-19 pandemic.^22^

Remote language services may bridge communication gaps for these patients, particularly in hospital settings where there are frequent brief interactions that affect the quality of care. Yet, implementation is critical in determining use and sustainability. Hospitals and health systems must engage users (including a wide array of clinicians as well as patients) in implementation and monitoring of remote interpretation to increase engagement and ensure quality and safety for hospitalized patients who prefer a non-English language.

Despite efforts to improve interpreter use across health systems and improvements in available technologies, interpreters remain underutilized in health care settings.^25,26^ Most interventions have resulted in modest improvements in language access.

Barriers to interpreter use are multifactorial, including cost and lack of reimbursement for interpreter services; quality of interpreters and lack of standardized training; clinician and patient beliefs about privacy, utility, and rapport building; clinician and patient skills and knowledge for working with interpreters; organizational culture and policies; underinvestment in infrastructure; lack of stakeholder input in implementation of interpretation modalities/devices; low resources for interpreter infrastructure; and challenging processes for acquiring an interpreter.^18,26^

Successful interventions will likely need multiple components to address these complex barriers to interpreter use. Interventions focused solely on increasing physician knowledge have been largely ineffective in achieving increased and sustained interpreter use.^18^ Similarly, it is not sufficient to solely increase interpreter devices in health systems, as evidenced by this dual-handset interpreter telephone intervention.^8,20^

Few interventions have targeted patients, with most focusing on increasing patient knowledge of existing language access services, rather than on patient-initiated interpreter use.^18^ The development of video-mediated interpretation may lower barriers to use and enhance the quality of interpretation, yet adoption of video-mediated interpretation in the hospital has been slow and variable.^24^

Our findings support some of the crucial components of multifaceted interventions to increase language access. To improve successful implementation of interventions aimed at increasing access to professional interpreters in hospital settings and to address the barriers that our participants highlighted, health systems should consider the following:

(1) Accompany intervention rollout with ongoing training, feedback, and reminders. Provide training in the proper use of remote interpretation and deliver consistent messaging on how professional interpretation enhances quality and safety for patients who prefer a non-English language. Periodic feedback and ongoing reminders to use professional interpretation, and how best to easily access those interpreters, may help to sustain long-term use. Our participants emphasized that use of telephone interpreters was most difficult to remember or use in pressured situations due to the perceived impact on time. Feedback and reminders may counteract decreased remote language service use over time.
(2) Encourage patient-centered care. Our participants highlighted that dual-handset interpreter phones are often clinician-centered tools. To ensure that patients as well as clinicians can initiate the use of remote language services, health systems can incorporate both patient and provider education. Future research should evaluate the effectiveness of such patient-centered approaches in increasing the use of remote interpretation services in the hospital setting.
(3) Perform continuous service quality monitoring. Clinicians noted that perceived past experiences with delays caused them to forego remote language services in subsequent situations with time pressures. To avoid this, once remote language services have been implemented, health systems can incorporate continuous tracking of professional interpreter use and the time needed to connect to interpreters, accompanied by quality improvement efforts to address any identified delays. Troubleshooting and corrective action to reduce delays can decrease the likelihood that clinicians will elect to forego dual-handset interpreter telephone use based on prior experiences with delays in service.
(4) Develop strategies to bridge communication barriers when remote language services may be inadequate. Our findings indicate that remote language services cannot fully replace in-person interpreters in all clinical circumstances or for all patients. Health systems can provide guidance to maximize appropriate use of remote language services and ensure other modalities are available when needed.

In conclusion, our results demonstrate that clinicians value dual-handset interpreter telephones in bridging communication barriers and highlight areas to guide future implementation of multifaceted interventions to increase the uptake of professional interpretation in hospital settings.

### Health equity implications

While more widely available in recent decades, remote interpreting modalities remain underused, placing patients with language barriers at risk for adverse events and suboptimal care. Our study highlights clinician barriers to access and use of dual-handset interpreter telephones, one widely available and persistent remote interpretation modality that can complement emerging technologies.

We suggest implementation strategies for multifaceted interventions to overcome these challenges in interpreter access for patients with non-English language preference in hospital settings; these recommendations should be tested and evaluated in future work.

## Availability of Data and Materials

The data used and/or analyzed during this study are available from the corresponding author on reasonable request.

## Ethical Declaration

All participants gave written consent to participate in the study. The study has been approved by the Institutional Review Board at the University of California, San Francisco. The study was conducted in line with research ethics based on the Declaration of Helsinki.

## Abbreviation Used

LEP: limited English proficiency

## References

[1] U.S. Census Bureau. Language Spoken at Home. American Community Survey. “2019: ACS 1-Year Estimates.” Available from: https://data.census.gov/cedsci/table?q=individual%20language&tid=ACSST1Y2019.S1601&hidePreview=false [Last accessed: February 20, 2021].

[2] Ryan C. The Limited English Proficient Population in the United States. 2015. Available from: https://www.migrationpolicy.org/article/limited-english-proficient-population-united-states [Last accessed: March 4, 2018].

[3] Agency for Healthcare Research and Quality. Chapter 1: Background on Patient Safety and LEP Populations. Available from: http://www.ahrq.gov/health-literacy/professional-training/lepguide/chapter1.html [Last accessed: December 15, 2020].

[4] Baker DW, Parker RM, Williams MV, et al. Use and effectiveness of interpreters in an emergency department. JAMA 1996;275(10):783–788. 8598595

[5] McFarland DC, Johnson Shen M, Holcombe RF. Predictors of satisfaction with doctor and nurse communication: A national study. Health Commun 2017;32(10):1217–1224. 27612390 10.1080/10410236.2016.1215001

[6] Ku L, Flores G. Pay now or pay later: Providing interpreter services in health care. Health Aff (Millwood) 2005;24(2):435–444. 15757928 10.1377/hlthaff.24.2.435

[7] Karliner LS, Jacobs EA, Chen AH, et al. Do professional interpreters improve clinical care for patients with limited English proficiency? A systematic review of the literature. Health Serv Res 2007;42(2):727–754. 17362215 10.1111/j.1475-6773.2006.00629.x PMC1955368

[8] Lee JS, Nápoles A, Mutha S, et al. Hospital discharge preparedness for patients with limited English proficiency: A mixed methods study of bedside interpreter-phones. Patient Educ Couns 2018;101(1):25–32. 28774652 10.1016/j.pec.2017.07.026 PMC5732033

[9] Flores G, Abreu M, Barone CP, et al. Errors of medical interpretation and their potential clinical consequences: A comparison of professional versus ad hoc versus no interpreters. Ann Emerg Med 2012;60(5):545–553. 22424655 10.1016/j.annemergmed.2012.01.025

[10] Divi C, Koss RG, Schmaltz SP, et al. Language proficiency and adverse events in US hospitals: A pilot study. Int J Qual Health Care 2007;19(2):60–67. 17277013 10.1093/intqhc/mzl069

[11] Lindholm M, Hargraves JL, Ferguson WJ, et al. Professional language interpretation and inpatient length of stay and readmission rates. J Gen Intern Med 2012;27(10):1294–1299. 22528618 10.1007/s11606-012-2041-5 PMC3445680

[12] Karliner LS, Pérez-Stable EJ, Gregorich SE. Convenient access to professional interpreters in the hospital decreases readmission rates and estimated hospital expenditures for patients with limited English proficiency. Med Care 2017;55(3):199–206. 27579909 10.1097/MLR.0000000000000643 PMC5309198

[13] Jacobs ZG, Prasad PA, Fang MC, et al. The association between limited English proficiency and sepsis mortality. J Hosp Med 2019;14:E1–E7. 10.12788/jhm.3334 PMC7064297 31891556

[14] The Joint Commission. 2019 Comprehensive Accreditation Manual for Hospitals (Edition). Joint Commission Resources: Oak Brook, IL; 2018.

[15] Diamond LC, Tuot DS, Karliner LS. The use of Spanish language skills by physicians and nurses: Policy implications for teaching and testing. J Gen Intern Med 2012;27(1):117–123. 21773850 10.1007/s11606-011-1779-5 PMC3250531

[16] Marshall LC, Zaki A, Duarte M, et al. Promoting effective communication with limited English proficient families: Implementation of video remote interpreting as part of a comprehensive language services program in a children's hospital. Jt Comm J Qual Patient Saf 2019;45(7):509–516. 31133535 10.1016/j.jcjq.2019.04.001

[17] Taira BR, Onofre L, Yaggi C, et al. An implementation science approach improves language access in the emergency department. J Immigr Minor Health 2021;23(6):1214–1222. 33387259 10.1007/s10903-020-01127-x

[18] Taira BR, Kim K, Mody N. Hospital and health system-level interventions to improve care for limited English proficiency patients: A systematic review. Jt Comm J Qual Patient Saf 2019;45(6):446–458. 30910471 10.1016/j.jcjq.2019.02.005

[19] Diamond LC, Schenker Y, Curry L, et al. Getting by: Underuse of interpreters by resident physicians. J Gen Intern Med 2009;24(2):256–262. 19089503 10.1007/s11606-008-0875-7 PMC2628994

[20] Lee JS, Pérez-Stable EJ, Gregorich SE, et al. Increased access to professional interpreters in the hospital improves informed consent for patients with limited English proficiency. J Gen Intern Med 2017;32(8):863–870. 28185201 10.1007/s11606-017-3983-4 PMC5515780

[21] Hsieh E. Not just “getting by”: Factors influencing providers' choice of interpreters. J Gen Intern Med 2015;30(1):75–82. 25338731 10.1007/s11606-014-3066-8 PMC4284281

[22] Diamond LC, Jacobs EA, Karliner L. Providing equitable care to patients with limited dominant language proficiency amid the COVID-19 pandemic. Patient Educ Couns 2020;103(8):1451–1452. 32571503 10.1016/j.pec.2020.05.028 PMC7304953

[23] Glaser BG. The constant comparative method of qualitative analysis. Soc Probl 1965;12(4):436–445.

[24] Hartford EA, Carlin K, Rutman LE, et al. Changes in rates and modality of interpreter use for pediatric emergency department patients in the COVID-19 era. Jt Comm J Qual Patient Saf 2022;48(3):139–146. 35058161 10.1016/j.jcjq.2021.11.003 PMC8590502

[25] Schulson LB, Anderson TS. National estimates of professional interpreter use in the ambulatory setting. J Gen Intern Med 2022;37(2):472–474. 33140275 10.1007/s11606-020-06336-6 PMC8811112

[26] Khoong EC, Fernandez A. Addressing gaps in interpreter use: Time for implementation science informed multi-level interventions. J Gen Intern Med 2021;36(11):3532–3536. 33948799 10.1007/s11606-021-06823-4 PMC8606497

